# Using a Smart City IoT to Incentivise and Target Shifts in Mobility Behaviour—Is It a Piece of Pie?

**DOI:** 10.3390/s150613069

**Published:** 2015-06-04

**Authors:** Stefan Poslad, Athen Ma, Zhenchen Wang, Haibo Mei

**Affiliations:** School of Electronic Engineering and Computer Science, Queen Mary University of London, Mile End Road, London E1 4NS, UK; E-Mails: athen.ma@qmul.ac.uk (A.M.); zhenchen.wang@qmul.ac.uk (Z.W.); haibo.mei@qmul.ac.uk (H.M.)

**Keywords:** smart cities, intelligent transport system, mobility behaviour, incentives

## Abstract

Whilst there is an increasing capability to instrument smart cities using fixed and mobile sensors to produce the big data to better understand and manage transportation use, there still exists a wide gap between the sustainability goals of smart cities, e.g., to promote less private car use at peak times, with respect to their ability to more dynamically support individualised shifts in multi-modal transportation use to help achieve such goals. We describe the development of the tripzoom system developed as part of the SUNSET—SUstainable social Network SErvices for Transport—project to research and develop a mobile and fixed traffic sensor system to help facilitate individual mobility shifts. Its main novelty was its ability to use mobile sensors to classify common multiple urban transportation modes, to generate information-rich individual and group mobility profiles and to couple this with the use of a targeted incentivised marketplace to gamify travel. This helps to promote mobility shifts towards achieving sustainability goals. This system was trialled in three European country cities operated as Living Labs over six months. Our main findings were that we were able to accomplish a level of behavioural shifts in travel behaviour. Hence, we have provided a proof-of-concept system that uses positive incentives to change individual travel behaviour.

## 1. Introduction

As smart cities prevail with an increasing capability to be instrumented with a diverse range of mobile and fixed sensors, connected via V2I (Vehicle to Infrastructure) V2V (Vehicle to Vehicle) and H2I (Human to Infrastructure) wireless and wired networks, these in turn enable a richer set of dynamic, open context-aware, IoT (Internet of Things) driven smart city ITS (Intelligent Transport System) services. This can be seen a key step towards a “Smart World”—an integration of smart environments to better understand our surroundings so as to improve our well-being [1]. Such services include adaptive personalised maps [2], adaptive vehicle navigation [3], smart fleet management and traffic monitoring [4], road incident detection [5], congestion avoidance [6], speed control via smart interaction with roadside controls [7], context-based vehicle maintenance [8], car parking aids [9], human driver monitoring [10] and better driving safety [11]. 

A main goal of many city and transport authorities is not just to operate effective public transport services and to manage the transport infrastructure effectively, but more strategically it is for its millions of users to undertake more efficient and sustainable urban travel. For example, in many cities, the use of petroleum fuel private cars often dominates journey choices by citizens. This in turn can cause serious environmental (e.g., greenhouse-emissions such as CO_2_, methane, NO_x_; noise, odour annoyance and acid precipitation), economic (e.g., use of non-renewable energy sources; and the time lost due to congestion) and societal (e.g., health problems such as cardiovascular and respiratory diseases; traffic casualties; community severance and loss of community space) repercussions [12]. The widespread development and use of more eco-friendly cars, that have a low carbon footprint, e.g., which emit 75 g/km or less of CO_2_ and that meet the Euro 5 standard for air quality, are still in their infancy. Hence, a more specific goal of transport authorities is often to reduce the use of private car use in cities and to promote a greater uptake in public transport use and human-powered travel, e.g., walking or cycling by offering travellers the right kind of incentives. An effective incentive is one that motivates individual travellers to change their travel behaviour so as to achieve the overall authorities’ goals, *i.e.*, to reduce car use during peak commute times, to protect the environment through reducing air pollution emissions, to promote well-being through using more human-powered mobility during urban travel and to facilitate personal travel safety. 

Our main research and development objectives are as follows: to analyse the types of incentives that can be used to shift citizen’s travel to meet specific transport sustainability goals; to research and develop an incentive system that is spatially-temporally aware and can target specific commuters in a spatial-temporal context either before starting or during their daily commute; to evaluate the use of a context-aware incentive system in multiple cities in groups of commuters. In order to achieve our research objectives, we highlight our main contributions as follows. First, our contribution is to meet these objectives. To the best of our knowledge, our novelty is that no current ITS system has been proposed and validated to meet these objectives (see Section 2). Second, our contribution is that based upon our design, implementation and validation experiences, we highlight some of the key issues to advance the use of targeted travel incentives towards improving travel sustainability goals in cities, specifically for road journeys of daily commuters (Section 5). The remainder of this paper is organised as follows. Related work is critically analysed (Section 2). The experimental framework is described (Section 3). The results and validation of the method are presented (Section 4). Finally, the conclusions are presented (Section 5).

## 2. Related Work about Travel Incentives

### 2.1. Scope and Focus

Although ITSs can be applied to several transport modes, our (research and development) scope covers the use of road and to an extent rail transport but not air travel or travel over water. In particular, we focus on transporting people not freight and the use of human-controlled rather autonomous vehicles. This focus is because the most dominant transport modes used within cities concern the road transport of people. An incentive can be defined as an “event or object external to the individual, which can incite action” [13]. Transport related incentives may take various forms, ranging from positive to negative incentives [14]. Positive incentives aim to promote the benefits of using that incentive for a particular transport mode such as cars. Car use can be reduced by altering travel choices and offering rewards. Examples of such incentives are park and ride, teleworking, transit improvement, and TDM (Travel Demand Management) marketing. In contrast, negative incentives focus on mechanisms that are disadvantageous if a specific transport use is not reduced. Therefore, most (if not all) pricing policies for car use fall into this category, such as fuel tax, parking fees, and congestion charges. Negative incentives such as congestion charges in urban areas have been proved to be unpopular with users and are seen to have a negative economic impact on local traders and residents. A survey of 2018 London, UK, residents between 1 September and 5 October 2008 found 41% wanted the London western traffic congestion charge zone abolished and 15% wanted to change the way the scheme operated [15]. A good overview of how incentives in general influence travel demand is given by Cairns *et al*. [16].

The remainder of this related work categorises travel incentives into four groups: real-time travel information, feedback and self-monitoring, social networks and rewards and points. Each of these is considered in turn. As smart phones are the dominant way by which travellers on route can access travel information any time, any place, the smart phone apps that can be used to deliver incentives will also be surveyed. The survey ends with a discussion.

### 2.2. Real-Time Travel Information

Providing people with information about the alternative transport modes is a common practice in a TDM marketing campaign. Such programs are designed depending on their target groups and the active involvement of key stakeholders, e.g., the involvement of employees and support have been identified as an important factor that determines the success rate of a TDM program aimed at commuters. The success rate of TDM marketing in reducing car use usually depends on participants’ predisposition towards public transport and only works properly when the infrastructure and services of the transport mode alternatives are already adequate [17]. With regard to the types of information involved, there are two groups of Travel Feedback Programs (TFPs): individualised and general TFPs. In the former case, participants are given certain information that they are asked for, whereas in the latter case, participants are given non-personalised information. Personalised information can be designed based on travel diary data provided by participants or data derived from a survey or interview. Users may also be provided with real-time information on the conditions of the transport network such as delays information, planned or emergency road works, park availability and traffic alerts and hazards (e.g., a road accident or a train cancellation). Information on these conditions can be coupled with personal habitual travel patterns to deliver information that are personalised to individual users’ information. Kramers [18] considers the user decisions that could lead to lower energy use and GHG (Green House Gas) emissions. Changes in travel patterns can be triggered and occur in any of the three different phases of the journey, pre-planning, on-route, post analysis. The choice of transport mode is dependent on the distance to the destination, what changes on-route from the norm and what users’ experiences are of their trips. To indicate if the individual’s choice leads to lower energy use, lower transport demand and the use of efficient modes of transport, the four-stage principle (FSP) from the Swedish Transport Administration can be used. This analyses: (1) Measures that reduce transport demand and transfer to efficient modes of transport; (2) Measures for optimized use of existing road network; (3) Minor road improvement and upgrading of existing infrastructure; (4) New investments and major road improvement [18] .

Real-time information is expedient because it accommodates changes that happen due to sporadic events on the road networks, e.g., delays, road works, and accidents. Current TFPs tend not to be able to deal with real-time travel-related information. Instead, there is a range of mobile device, including Smart Phones and more dedicated devices such as SatNav, apps that offer real-time information such as an alternative route on a currently congested route, weather *etc.* Some real-time apps have also been given in the introduction. Two useful surveys of real-time travel apps for mobile devices are included as part of more general survey on smart city sensing [19] and on mobile phone sensing [20]. One specific example of a mobile app to promote greener transportation use is UbiGreen [21]. This system was limited to a three week field study with 13 users. At the end of the study, an open ended response exit questionnaire was used to ask whether participants felt that UbiGreen had encouraged them to travel in a more ecofriendly way. There were too few responses to draw meaningful conclusions.

### 2.3. Feedback and Self-Monitoring

TFPs (Travel Feedback Programs) provide personalised feedback on individual travel behaviour. These aim to provide travellers with tangible evidence in a comprehensive manner, so as to raise their awareness on impact caused by the way they travel. The metrics used are often relatively simple, such as cost, time, calories, distance and carbon footprint. This functionality provides a way for users to monitor their own behaviour. Recommendations can also be suggested to users based on their performance and by identifying suitable alternatives. Fujii and Taniguchi [22] classified TFPs according to place, technique, procedure, and communication media, and reviewed the effectiveness of 10 TFPs in Japan. They found that TFPs reduced CO_2_ emissions by about 19% and car use by about 18%, while increasing the use of public transport by about 50%. In addition, they found that TFP effectiveness increased when participants were asked to make behavioural plans to change their travel habits. Rose and Ampt gave participants customized tips on “how to” reduce their car use and let them exercise on these tips [23] whereas Tertoolen *et al.* focused on making people aware of the consequences of their travels on costs and CO_2_ emissions [24]. Findings of these studies indicate that self-monitoring could be a useful tool to help people reduce their car use because it conveys people’s travel behaviour into more tangible forms, e.g., total costs, distance, time, calories, and CO_2_ emissions. However, alone self-monitoring may not yield satisfactory results. Self-monitoring should be tailored to other customized information, such as travel information, tips, and mobility coaching on how to reduce car use.

There currently exist a range of apps that can be downloaded from any of the major smart mobile phone vendors’ marketplaces that let users set their own targets for travel, such as CO_2_ reduction and weight lost and that help users achieve them through tips and monitoring. Self-monitoring one’s own travels, combined with setting travel targets and individualised tips seems an interesting incentive type to reduce car use. However, there is a lack of scientific analysis of how effectively mobile travel apps work.

### 2.4. Social Networks

The key incentive of social networks for travel is to provide users with a means to communicate, and to share their travel experience and information with each other. In doing so, there is a strong evidence that allowing users to share their performance is a way to: boost their achievement and trigger competition; help promote group behaviour; increase trust among users and reduce social ambiguity [25]. Cho *et al.* [26] show that social relationships determine about 10% to 30% of all human movement, while periodic behaviour determines about 50% to 70% of human movement. Whereas Batterbury [27] shows that a key element for more sustainable transport alternative requires strategies that engage active cooperation between the key local stakeholders rather than instigating radical new initiatives. Binsted & Hutchins [28] conducted a study with 22 participants to investigate the role of social networks in changing people’s travel behaviour in the UK, focusing in particular on sustainable transport. Only around 20% of the participants have used social network sites to access information related to public transport, and even less have used social networks to actively make decisions based upon this information, e.g., 15% have arranged car sharing. Privacy is a main concern for social networks and affects the way in which data are stored and for what period of time. Anonymity and disclosure of data must be treated with the greatest care [29].

### 2.5. Rewards and Points

There is a large number of computer and smartphone games that use a points based system to motivate players to stay “online”. The rationale of using points as a form of reward can be explained that an activity that is easy to do (such as playing games) will require only little motivation for a person to finally commit to it [30]. Thus, triggers such as points may be enough to drive a person to play a game. However, when an activity is hard to do, it will require much stronger triggers. Travel can be gamified using a points based system. However, changing people’s travel behaviour can be a difficult task, especially when there are many factors which influence one’s behaviour, such as constraints in daily activity scheduling. Therefore, linking bonus points to different rewards may be a necessary step so as to provide the extra “push” required to get travellers to change their behaviour.

Rewards and punishments are examples of extrinsic motivation, defined as external factors outside individuals that aim to encouraging people to accomplish a goal. Both rewards and punishments are also regarded as methods to discipline people, making them retain the promoted behaviour. Rewards are varied from simply giving a verbal compliment to monetary reward. Likewise, punishments also have a wide range, from verbal warning to fines (e.g., speeding ticket) and social exclusion. Several behavioural transportation studies have found that reward strategies are generally more successful than punishments [31,32]. 

While the ineffectiveness of punishment to support a lasting behavioural change has been verified by many behavioural studies to date, the effectiveness of rewards is still a subject of debate; rewards can lead to effective results but may be only for a short period of time. Cameron and Pierce [33] argued that rewards can be used to motivate and maintain people’s self-interest in doing certain activities. In contrast, Deci [34], Harackiewlcz [35] found that rewards, in fact, reduce people’s intrinsic motivation. Intrinsic motivation is defined as motivation linked to people’s innate psychological needs, such as senses of curiosity and exploration. This can also be motivation derived from the activity itself. Deci [34] argued that intrinsically motivated behaviour requires no reward because it is performed out of interest and enjoyment. 

### 2.6. Discussion

Gamification of travel coupled to the use of incentives can encourage travel-related behavioural changes during commuter journeys. Incentives can be classified in terms of four categories of real-time travel information, feedback, social networks and points and rewards. Several studies have shown that providing people with personalised travel information could eventually alter people’s travel decisions, for instance with regard to mode, route, and departure time choices. This could be strengthened by providing this information in real-time. It has also been shown that travel information could take various forms, namely: real-time personalised travel information about road networks, such as delay information, road works, and parking availability; Real-time personalised travel information about transport mode alternatives, such as (the closest) bus stop and departure time information.

Feedback as intrinsic motivation may have the ability to alter people’s travel behaviour and retain the changes in the long-term as it allows people to monitor their own behaviour, letting them control their own travel choices. For example, travellers can see the impact of their travel behaviour on the environment, e.g., in terms of CO_2_ emissions, and on themselves, e.g., in terms of calories, costs, and time. Feedback appears more useful for those who, to some extent, have already positive attitudes towards the environment, health, or costs, but may work less well with others whose attitudes are more negative. Empirical work should test this incentive further to find out about users’ preferences towards self-monitoring combined with the possibility to set personal travel targets, e.g., related to time, cost, distance, CO_2_ and calories.

Rewards such as points as incentives have mixed results. Tangible rewards, such as monetary rewards, can reduce people’s intrinsic motivation but these incur a substantive economic cost and it is unclear if this cost can be sustained. Non-tangible rewards, such as games points need further investigation by studying potential travellers’ attitudes towards this incentive type, in particular related to points when performing “good” behaviour, and possibly linking this to tangible rewards to redeem the collected points.

Social networks can be used to alter people’s travel behaviour due to peer influence. Thus, social networks can be used as a mechanism to enhance other incentive categories, e.g., a system could suggest to users to cycle, walk or to take public transport together with others who have similar travel patterns. Social networks seem a very useful medium for people to exchange tips and experiences, such as tips related to certain modes or routes.

## 3. Method 

To the best of our knowledge, the use of a targeted incentive service in combination with mobile phone sensing of transport modes and coupled to social networking has not been proposed or examined in depth to date in order to empower individual users to travel more sustainably. The design and implementation of a system called the tripzoom is described in this section, Section 3. It is evaluated in the next section, Section 4, and is discussed in the final section, Section 5, where conclusions concerning the development and users’ evaluation experiences are drawn.

### 3.1. Requirements and Development Methodology

The user requirements for the EU FP7 SUNSET Project system called tripzoom are based upon a structured analysis of the use-cases. The process to analyse the scenarios to derive the user requirements is as follows:

- Analyse the scenarios expressed in natural language.
- Analyse the scenarios for functional *versus* non-functional requirements.
- Analyse the scenario description in order to derive user requirements.
- Review that the parts of the complete scenario are all high priority to be developed for the Living labs (LL) trials (see Section 4).
- Review if all major system components requirements are used in the scenarios via analysing that that are then mapped to the system requirements. This confirms how the system supports the user requirements.

The system requirements to support the use-cases are grouped into the following service blocks (see Section 3.2): mobility server sub-system, mobility client sub-system and Infrastructure network and portal sub-system. In more detail, 95 system requirements for the mobility server were identified: 11 for the Personal Mobility Store, 13 for the Mobility Pattern Detector, 9 for the Mobility Pattern Visualizer, 13 for the Incentive Market-Place, 10 for the Experience Sampling Store, 16 for the Relation and Identity Manager, 11 for the Privacy Manager, 5 for the Evaluation Support and 7 for the Infrastructure Network Manager. Most system requirements have a strong link with the scenarios, but the list also includes a few system (non-functional) requirements without a direct link to the use cases.

Table 1 shows the mapping of how user requirements derived from use-cases are related to tripzoom components. There are also some functions not realised because of strategy changes during development. A few are not supported as the focus shifted to be more heavily reliant on personal mobility rather than on integration of real-time traffic data, and the focus shifted from a traveller’s access via a PC-based Web portal and mobile phone to the use of mobile phones only to access services. A reverse mapping of system component functional requirement to user functional requirements was also performed as a cross-check. For each system component, the detailed functional requirements were also tabulated. An example of some of the requirements for the IMP is given in Table 2.

**Table 1:** Exemplar user requirements mapped to system requirements (RIM—Relation and Identity Manager; MPD—Mobility Pattern Detector; PMS—Personal Mobility Store; INM—Infrastructure Network Manager; MPD—Mobility Pattern Detector).

Scenario ID	User Requirements	System Component & Requirements	Priority
US2	Social Network Reuse	RIM.1 (identify and authenticate user), RIM.3 (link Social Network user ID to tripzoom user ID), RIM.5 (import social network links into tripzoom)	High
US4	Improved Mobility Pattern Analysis	MPD.1 (create mobility patterns for persons, places & vehicles), MPD.4 (provide overviews of mobility choices of users & consequences of choices, e.g., time, money, emissions, delay), PMS.2 (pre-filter sensor data), INM.4 (Get mapping of location trace to a list of infrastructure segments); INM.5 (reverse geo-coding and mobility-related Point of interest lookup).	High
US8	Planned Real-time Trip Info & Recommender	MPD.3 (classify context-aware mobility patterns where context can be weather condition, or user-event driven, e.g., shopping)	High
US9	Real-time Trip Info.	MPD.1 (as above)	Medium
US13	Trip Change Incentives	PMS.8 (pre-filtered sensor data exchange), IMP.4 (offer context-aware, incentives e.g., in space and time, to targeted users)	High
US14	Ad hoc Location-specific Mobility Offers	IMP.4 (as above)	Medium

**Table 2:** Exemplar IMP (Incentive Market-Place) system component requirements where SS represents the transport authority stakeholder.

Component	IMP (Incentive Market-Place)			
Responsible	Named Consortium Members			
Number	Description	Source	Rationale	Priority
IMP.1	Provide system’s definition of an incentive in terms of “Who, When, Where and How” so as to allow providers to publish/update/remove incentives accordingly.	Technical	System’s definition on incentives.	High
IMP.2	Provide a bonus point based reward system in which users can subscribe to.	SS3	Platform where incentives are managed; this decouples issuing incentives from issuing rewards.	High
IMP.3	Manage the different types of users of the system: incentive providers and system users.	Technical	This is required for general operation.	High
IMP.4	Offer incentives to users by taking into consideration their mobility patterns, identified potential behavioural changes, preferences, circumstances and history of incentive scheme participation so as to achieve system’s and individual’s goals. Incentives are context-based, e.g., depend on time and place.	US13, US14	Offer incentives with reference to individuals’ travel patterns.	Medium

An iterative, interactive and formative, development approach was adopted to deliver the complexity of the tripzoom system in a managed way. Seven major iterative releases of the tripzoom system were produced in the second year of a three-year project. Earlier releases developed the simpler core system functionality and later releases extended this functionality, and on occasion, maintained existing functionality from a previous release. The main system releases with respect to the user requirements and services are as follows:

- Release R1: focussed on users’ access to the tripzoom system services;
- Release R2: focussed on user-oriented (privacy) access control to services and to richer core travel information services;
- Release R3: added the first support for incentives and for experience sampling;
- Release R4: improved the travel information services, use of incentives and the use of social network services;
- Release R5: focussed on: improving the use of incentives, user control of privacy preferences and on richer information views and on transport authorities or city moderators being able to first sample users to evaluate experiences.
- Release R6: focussed on better supporting city authorities to be able to issue experience sampling, to compare mobility patterns of user groups and to get overviews of grouped trips.
- Release R7: focussed on improvements in the service design of R1 to R6 and to offer more support for information management with respect to more finely-grained groups of travellers.

The development and testing of each major release of the system took place on average every six to seven weeks, including a one to two week user evaluation with a limited set of factors internal to the project non-developer users; external user evaluation is given in Section 4. This led to a formative evaluation of the user requirements and design rather than a summative evaluation to validate the finished design. This enables the evaluation of a preceding design to help form a better proceeding design. During the iterative development and formative evaluation of the system, the view of the system shifted from a user-requirements driven system to a user-centred service-oriented (S-O) type system. This was driven by several needs:

- Often users assume but do not explicitly state some core non-functional system functions (implemented as services) such as privacy, help and language localisation to support international users’ use of different languages and country contexts. An S-O system also captures and models these implied ‘support’ user services.
- Systems tend not to be designed and implemented in terms of high-level user requirement driven sub-systems but in terms of more technical, reusable, lower level (atomic and composite) service components. Hence, in the second year of the project, the development of the system focussed on the S-O view rather than on the user-requirements *per se*.
- During the iterative development, it became clearer which user requirements could feasibly be realised using system services in an operational system.

The S-O view analysis also identified the following user roles: Traveller—User of the tripzoom services; City authority or moderator—Operator and controller of the tripzoom services in each LL city; Third party—service providers who offer travel related services, who may provide a means to exchange physical rewards for incentive points, e.g., offer a free piece of pie or a discount on buying a beverage near a public transport station based upon the use of public transport to that station; Researcher—can provide information to their user community on the LL goals, approach and privacy aspects.

### 3.2. System Overview

**Figure 1:**
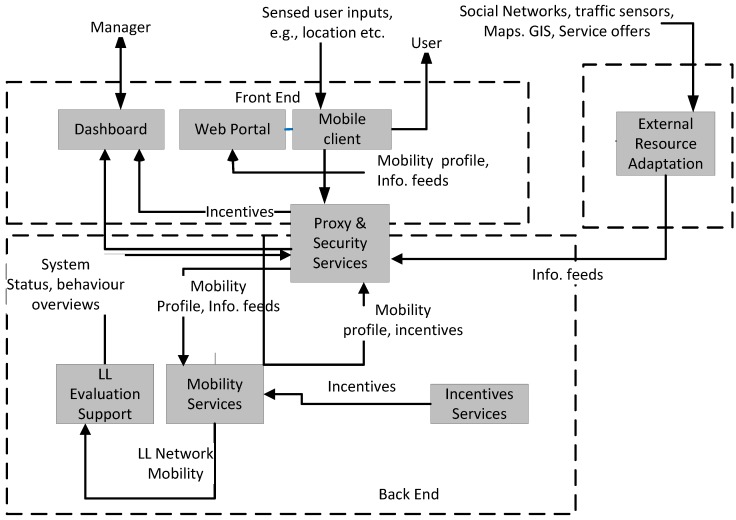
High-level architecture for the tripzoom system.

The high-level design of the architecture as a distributed system is based upon a client-proxy-server model. The main sub-systems that comprise the overall system are shown in Figure 1. A hybrid distributed system interaction style is used by the system to support loose-coupling between system components. The system combines the use of Representation State Transfer (REST) Models, Shared Data Repository (SDR) and Event-Driven Architectures (EDA) for different parts of the system. Different kinds of state information can be exchanged as metadata when one process invokes another. In the Representation State Transfer (REST) model, the receiver is seen as a set of resources identified by URLs. Loose-coupling is supported in that only representations of the resources are exchanged, not any state information. This can for example be used to support the experience sampling interaction. In a SDR, data producers store data and can be operated independently from data consumers that can modify and read the data, e.g., mobility sensing can act independently from mobility pattern detection. 

In an EDA, events are generated by event producers. They are gathered and distributed by an event dispatcher to any event consumer that wants to be notified of them. The event consumer can perform its own checks if events meet certain constraint conditions and if they do, one or more actions or services are triggered. The event producers are loosely coupled to the event consumers. These clients, services and proxy sub-systems are comprised of individual system components (see Table 3) at a lower level. 

**Table 3:** Classification of the main SUNSET system components.

Sunset Sub-Systems	System Components
Mobile Client	Mobile Experience Sampler, Mobile Incentive Presenter, Mobile Mobility Profile Visualisation, Mobile Buddy List, Mobile Notifications Mobile Authentication, Mobile Sensing
Web Portal (User)	User & Application Management, Portal Profile Visualisation
Dashboard (Manager Web Portal)	Living Lab Control & Evaluation
Proxy & Security Services	Privacy Manager, Relation and Identity Manager, Proxy & Authentication
Mobility Services	Personal Mobility Store (PMS), Mobility Pattern Detector Mobility Pattern Visualizer, Traffic Pattern Detector
LL Evaluation Support	Experience Sampling Store, Evaluation Support
Incentive Services	Incentives Market Place (IMP), Incentive Simulation Environment
External Resource Adapters	Social Network Adapter, Infrastructure Network Manager, Infrastructure Status Store, Weather Information Adapter

The mobile client runs on the mobile access device used by the end-user. Two main access devices were supported and differentiated by operating system, Google’s Android and Apple’s iOS. The mobile client consists of several sub-services. The ones pertinent to the operation of the Incentive service are as follows. A MS (Mobile Sensing) service uses the GPS mobile phone sensor to determine location and from this derive the speed. Locations tracks were also mapped to public transport routes defined in a GIS (Geographical Information System) database to help identify if tracks could be classified as public transport routes and modes. The MS also activates, deactivates and adjusts the available sensors and the sampling rates to optimise energy consumption as well as data quality. The MS stores sampled data on the device to the extent needed to fulfil its tasks and periodically sends it to the PMS (Personal Mobility Store). More details of the tripzoom MS service are given in [36]. The Mobile Incentive Presenter (MIP) provides the mobile interface of the IMP. It presents incentives to the user and sends the users responses and acceptances back to the IMP. The communication between the MIP and the IMP utilises the Mobile Notifications mechanism. The Mobile Notifications component receives notifications from tripzoom coming from the Experience Sampling Store (ESS), Incentives Market Place (IMP) or Mobility Pattern Detector (MPD) and dispatches them to the mobile client component responsible for processing a given type of notification. As such this component allows the platform to send messages to the mobile users or to the mobile application, for example to influence the location-sampling rate based on historical information computed on the server-side.

The (User) Web Portal consists of the User & Application Management (UAM) component provides the front-end of the RIM to users and developers. This allows developers to register external resources and applications together with a specification of the required access patterns and individual users to grant and revoke registered external resources and applications with respect to specified access rights. This also consists of the Portal Profile Visualisation (PPV) component to provide the front-end of the Web Portal for the users of the tripzoom system. The Dashboard (Manager Web Portal) consists of the Living Lab Control & Evaluation (LLCE) component that is used to monitor the flow of data in the SUNSET system by means of a number of visualisations (e.g., usage statistics, behaviour overviews) and analysis tools (e.g., effectiveness of incentives, results of experience sampling services). The Proxy & Security Services consist of the Privacy management component that manages the privacy policies of the user. Those policies are formulated based on social relations managed by the Relation and Identity Manager (RIM) or ad-hoc groupings computed by the MPD (Mobility Pattern Detector). It then decides on the access to the mobility data when asked by the policy enforcement point of a personal data handling components such as the MPD or the PMS. 

The Mobility Services consist of the following service components: the Personal Mobility Store (PMS) collects all raw measurements from the mobile client, and provides methods to enrich these measurements for the purpose of pattern detection and experience sampling; the Mobility Pattern Detector (MPD) receives data from the mobility monitoring components, such as the PMS (Personal Mobility Store) plus external sources; the Mobility Pattern Visualizer (MPV) takes the patterns derived by the Mobility Pattern Detector (MPD, Section 2.7.2) and turns them into interpretable and easily accessible visualisations; the Traffic Pattern Detector (TPD) component processes the infrastructure data from the Infrastructure Status Store (ISS). The LL (Living Lab) Evaluation Support describes the Living Lab Evaluation-specific server-side components of the SUNSET system. It consists of: the Experience Sampling Store (ESS) allows researchers to register questions for a specific target group in certain context conditions; the Evaluation Support (ES) component collects and collates information from other system components for the preparation of performance evaluation of the overall system.

Several information resources are considered to exist outside and to be external to the SUNSET system. SUNSET includes resource adapters to incorporate these within SUNSET. Most of these adapters are localised or location-specific such as the Infrastructure Sensor, Network Manager, Weather Information Adapter and Social Network Adapter. The latter interfaces with several well-known social networks, in order to facilitate user registration based on existing accounts to provide services to share information, such as successfully completing an incentive, with others, and to bootstrap the process of creating a buddy list by importing contacts from existing social networks.

The Infrastructure Network Manager (INM) provides a collection of services allowing access to external information such as weather, road networks and their characteristics, public transport information, transport routing services and other geographical data, such that personal mobility information can be mapped to these information sources. The ISS provides persistence for the INM, e.g., road works, traffic jams, speed traps and travel times for the purpose of routing and travel time prediction. These are situated in the external resource adaptation sub-system because fixed transport network sensors are envisioned to be different for every Living Lab, even although a common adapter model is used to integrate these sources into tripzoom. The incentive services are described in the following subsection.

### 3.3. Incentive Market Place (IMP)

**Figure 2:**
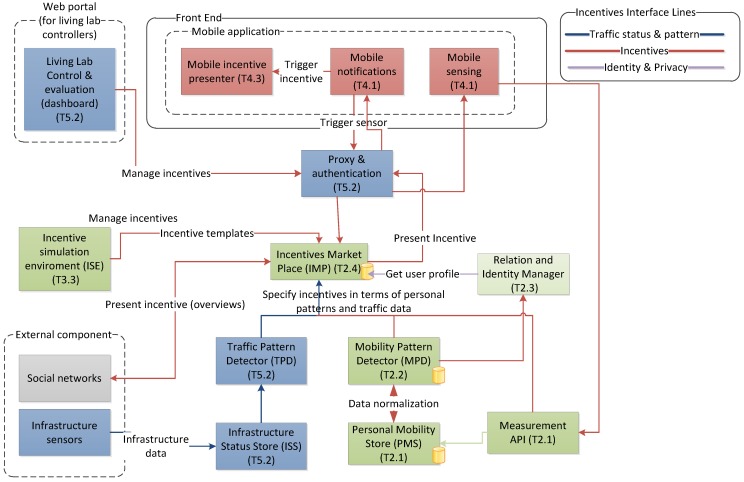
System interaction to support incentives distribution (the database symbol at the edge of some components indicates that that component is associated with an internal data store; T identifies the part of the project, the task, that developed that component).

The system interaction to support incentives distribution is given in Figure 2. The core part of the IMP matches the right incentives with the appropriate (groups of) users and delivers these when travellers at appropriate places and times without bombarding the users with different offers. 

The Incentive Market Place (IMP) provides a platform to offer incentives in the form of rewards, recognition, and real-time feedback to users to encourage travellers to improve their travel behaviour with respect to the system’s and an individual’s travel objectives, e.g., for sustainability. The IMP implements algorithms that match the available incentives with the mobility information from the MPD and with the individual user preferences and general transport information from the ISS. Users whose travel behaviour ought to be changed and the segments of the journey that could be optimised are identified. This is first based upon classifying the acquired mobile sensor data of each user to derive location, classify their transport modes and to determine which travel mode is used at peak time. Second, transport route information is also selected based upon aggregating similar or repeat tracks for a user, trip start and end locations and the public transport route options available between trip starts and ends. Based upon the personal mobility patterns and this route information, the IMP is able to offer right incentives at right times to users via a mobile client. The IMP also records users’ responses to the incentive offers and calculates the overall participation rates. Via Web APIs, incentive providers can register and publish incentives via the IMP.

The Incentive Simulation Environment (ISE) provides an environment to test the effectiveness of incentives on historic data in the tripzoom system. In such a way, both stakeholders and developers can investigate how the incentive can be tailored to target the users they wish to address without introducing them to the system just yet.

The Mobile Incentive Marketplace (MIM) is designed to support the delivery of incentives to travellers so as to promote sustainable travel behaviours. There are four types of incentives provided by tripzoom, namely, traveller mobility pattern, targets and challenges, loyalty points and social networks, which were analysed in the survey (Section 2). The MIM is designed to focus on delivering incentives in the forms of targets and challenges and loyalty points by referring to users’ travel routines, related to the conditions of the transport network of interest. The MIM is the key component in delivering incentives in tripzoom, and its operation is dependent on Incentive MarketPlace (IMP), in which incentives are registered, managed and monitored. A key feature of the IMP is its ability to examine a user’s mobility information, his or her preferences, and to identify a target or challenge that would help the user to achieve a certain goal, such as to avoid travelling in rush hour. The MIM is designed to optimise the delivery of such target or challenge in terms of the time and the right users. In addition, the MIM acts as a gateway between the users and Tripzoom, as users’ responses to an incentive is directly fed back to the system via this module, providing crucial statistics for the evaluation of the incentive of interest. The overall features of the MIM can be summarised as follows:

(1) Incentive management
  - To release and notify mobile users new incentives
(2) Monitoring travellers
  - To release and notify mobile users new incentives
  - To monitor historical trips status including travelling cost, CO_2_ emission, distance travelled and transport modality
  - To examine travellers’ incentive execution status
  - To assess travellers’ reward status
(3) Issuing rewards to travellers
  - To compare traveller’s behaviour with reward criteria
  - To send reward notifications to users

**Figure 3:**
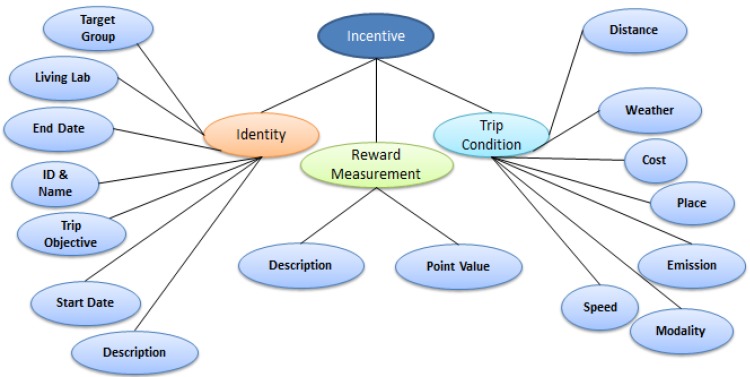
Incentive Data Model.

Data models in the Incentive Marketplace can be categorised as incentive-centric and user-centric. Both types of data models provide the criteria used to define an incentive. Figure 3 shows an overview of the Incentive data model, and an incentive in the form of a target or challenge is described by the identity, reward measurement and trip condition modules. The identity module provides definitions of a target or challenge, ranging from the intended target groups of users and living lab of interest to start and the expiry dates of such incentive. The reward measurement module provides descriptions of individual targets and challenges, and keeps track on the value of each of the associated reward. Finally, the trip condition module stores metrics that describe individual trips, such as the distance measured and modality detected for a trip. 

Communication between the MIM and a mobile client is two-way and messages are exchanged via HTTP using RESTful Services. The MIM sends the following messages to a mobile client

- Notification of a new challenge.
- Notification of a reward upon completion of a challenge.
- A list of current challenges applicable to an individual user.

A mobile client sends the following messages to the MIM

- Trip information of an individual user
- A request to find out all the current challenges applicable to an individual.

Matching the right group of users with the right target or challenge is a critical process that takes place inside the IMP. Such a process is semi-automatic, offering the flexibility for a living lab coordinator to define a target group as well as an appropriate target or challenge with respect to specific requirements.

1. *Target/challenge validity check*. This is to check whether a specific target/challenge is active; expired targets and challenges will not be considered further.
2. *Living lab identification*. Subdivide Tripzoom users by allocating them to the corresponding living labs: Enschede, Gothenburg and Leeds.
3. *Target group identification*. User can be further divided into subgroups by referring to a range of available demographic properties including gender, age, home city and household status.
4. *Trip modality matching*. A target and challenge is often designed to change a user’s mode of transport based upon modality information of a user obtained from the PMS and will use the modality requirements set for a specific target and challenge. A reward will be issued if the matching result shows that the user has fulfilled the given target and challenge.

**Figure 4:**
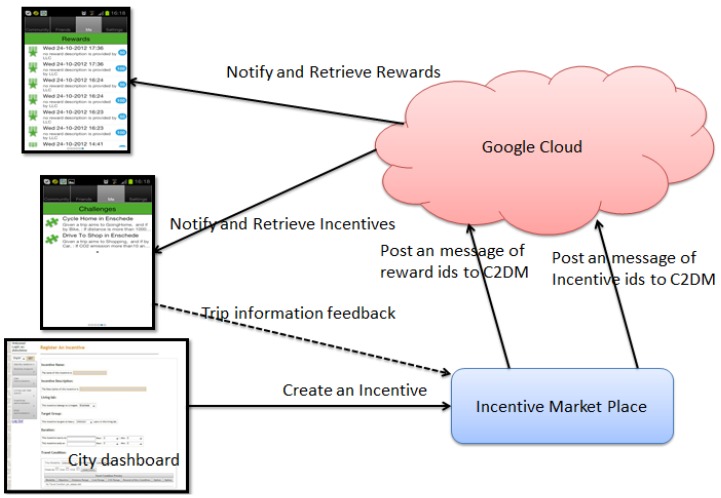
Distributing challenges and rewards to the Android tripzoom app.

A multi-component life cycle is in place to distribute target and challenges as well as loyalty points to users, as shown in Figure 4. The IMP is a gateway between the application Tripzoom and the city dashboard which is the control interface for the living lab controllers. The IMP communicates with the mobile client through the Google Cloud for the Android implementation, and in a similar fashion using Apple Push Notification Services (APNS). 

**Figure 5:**
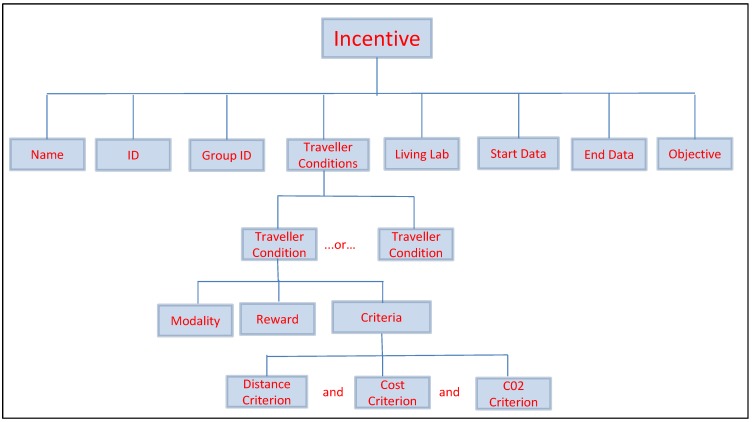
Structure of a “Target and Challenge” Incentive.

To manage incentives, the CD (City Dashboard) retrieves and sends incentive data from and to the IMP. LLc (Living Lab [human] controller) can register, retrieve, and modify incentives in IMP database. In addition, the CD supports LLc monitoring and incentive issuing to users, for example how many users received a new incentive notification. In total, there are two main tasks on incentive management now in the CD: registration and modification of incentives; monitoring incentives. The IMP currently implements “Target and Challenge” incentives. These incentives are manually registered by a LLc through the CD. The structure of an incentive is displayed in Figure 5 below. 

As shown in Figure 5, each incentive has several Traveller Conditions. In practice, if a user satisfies any one of the traveller conditions when finishing his or her trip, he or she could be allocated a related reward by the LLc. To reward a user, each traveller condition retrieves the specific modality (walking, bike, bus, *etc*…) and if satisfies part or all of the distance criterion, cost criterion and CO_2_ emission criterion. For example, three traveller conditions for an incentive might be:

- If you go by foot and travel more than 5 km, you will receive 100 points as a reward;
- If you take a bus and it costs less than 2 Euros and emits CO_2_ less than 5 mg, you will receive 50 points as a reward;
- If you take a car and travel less than 8 km and it costs less than 5 Euros and emits CO_2_ less than 10 mg, you will receive 20 points as a reward.

According to the three traveller conditions given above, if a user walks 6 km in a trip, he or she will receive 100 points. Another example is if a user takes a bus and spends one Euro and emits CO_2_ of 4 mg in a trip, he or she will receive 50 points. Yet another example is if a user drives a car and travels 10 km in a trip, then he or she will receive no points from this incentive even though he or she spends less than 5 Euros and emits CO_2_ less than 10 mg. 

These incentives are implemented as stored data objects in a relational data management server (Microsoft SQL Server). The conditions or rules for the incentives and the conditions or rules for associated rewards are implemented as stored procedures in the database accessed via a Restful Web Service interface at the City Dashboard user interface.

**Figure 6:**
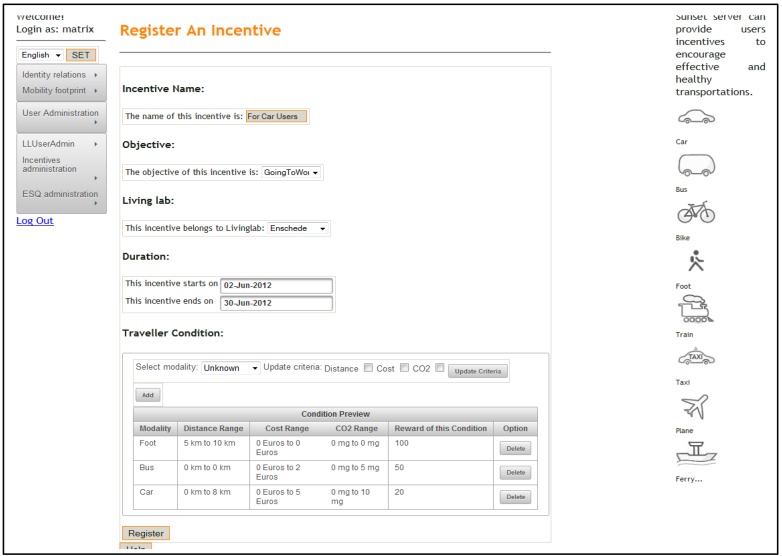
Incentive registration.

An example of an incentive configured by a LLc and registered in the CD is shown in Figure 6, This follows the incentive structure shown in Figure 5. After registration, an incentive can also be modified by a LLc. A LLc can visit the incentive monitoring page to see all the incentives from the IMP including the ones created by other LLcs. Each incentive in the list is organised following the incentive structure given in Figure 5. LLc can navigate, sort, filter and search through the incentive list. To monitor incentive issuing to users, there is a “Check” button to check how many users are notified by each incentive. The feedback page is provided once a user clicks an appropriate button. To monitor users’ response after they have been notified of a new incentive becoming available, the CD provides functionality to check how many users confirm each incentive after notifications are received. The feedback page is provided once the feedback button is clicked.

**Figure 7:**
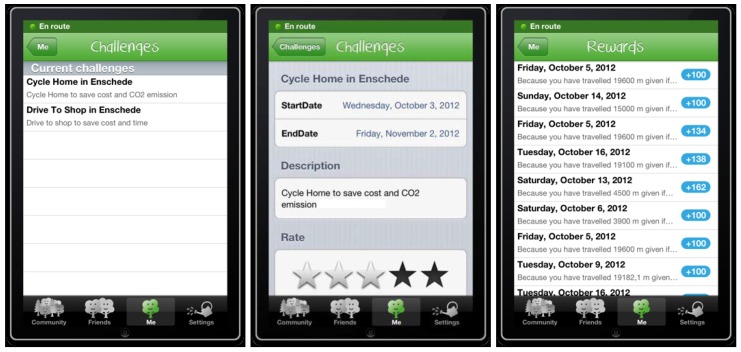
Challenges and Rewards.

An example of challenges and rewards offered by tripzoom for the Android app to a user is given in Figure 7. The challenges for an incentive (left and middle screen in Figure 7) describe the task that a user needs to do. The rewards given to users who manage to succeed with their given challenge, as detected by monitoring the location track, time and detecting the transport modes used in their trips, are shown Figure 7 (in the right screen ). For a user, the dynamic set of challenges is characterised and represented by:

- A title and a description, providing details about how to achieve the task set by the challenge;
- A time period in which the challenge is open;
- Rating of 0–5 stars showing the average rating that this challenge was given by the community as each user who received the challenge can rate it.

As soon as a challenge has been accomplished, the user is given a reward; for the user, a reward is characterised and represented by:

- The date and time it was received;
- An explanation as to why it has been received.
- A value representing the number of points the user has been awarded with this reward.

### 3.4. Deployment Outline

**Figure 8:**
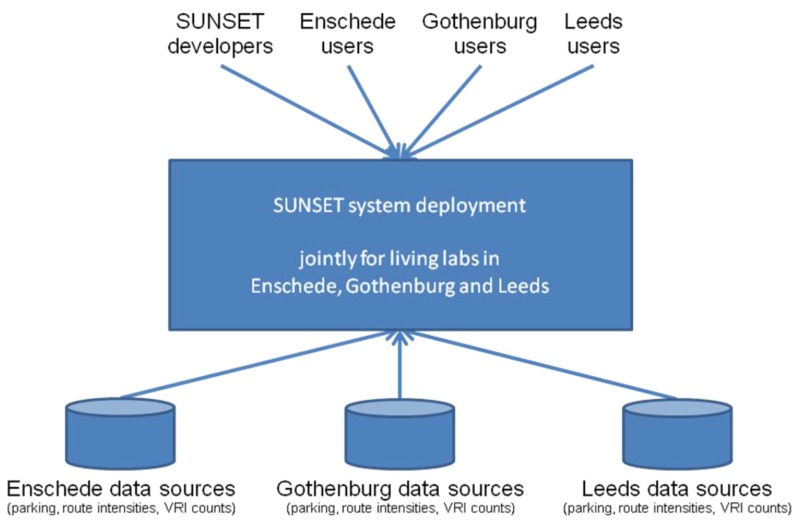
SUNSET deployment outline.

To simplify platform monitoring and reduce maintenance times, we used a single deployment that is shared between all the living labs. An outline of such a deployment is provided in Figure 8. The various living lab specific data sources are situated at the bottom, providing their data to the joint tripzoom system deployment. End users can use the web browser or mobile application to interact with the platform. This also provides to developers, e.g., to get an overall view of the system status, to adapt the configuration, or to deploy new components or register new applications. 

## 4. Evaluation

### 4.1. Technical Trials

For tripzoom iterations or releases 1 up to 7, these were only released internally to users associated with members of the project. The method selected for the internal evaluation was a hybrid heuristic evaluation and cognitive walkthrough. Both are commonly used usability inspection methods. Each release identified issues that were addressed in the next release. The internal evaluation results highlighted two major technical hurdles for the development of continuous mobility sensing apps like tripzoom. One is the energy-efficiency of the location sensing and the other is the accuracy of general location sensing and modality detection. In general, accuracy can be traded for energy-efficiency to some extend as we indicated above. 

### 4.2. User Experiments 

For release 8, tripzoom was released to user recruited within SUNSET three LLs in Enschede, Gothenburg and Leeds. LLs have been designed, organised and maintained for nearly a year in the 3rd final year of the project. Tripzoom and the incentive schemes delivered via tripzoom are evaluated in the LLs using a comprehensive evaluation methodology. 

First, people were recruited via a variety of methods such as flyers, press releases, social networks, and invited to join the SUNSET project and install the tripzoom app. Participants were then provided with more information about what the project entails and what to expect over the course of the LL. Registered were give some time to become familiar with the tripzoom app and for tripzoom to try to identify their personal mobility patterns and routes (up to two weeks). The characteristics of users recruited and their use of tripzoom for the evaluation experiments are given in Table 4.

**Table 4:** Characteristics of users recruited for the tripzoom evaluation.

LL City Location	Enschede, NL	Leeds, UK	Gothenburg, SE
No. Participants	268	112	138
No. tripzoom users	108	6	95
No. of recorded trips	28104	2157	19,746
No. KM traveled in trips	355874	19673	337698

In the second phase, the participants were subjected to focused experiments. These experiments were designed by a LLc to address one or more of the objectives of SUNSET. An appropriate target group was selected out of the participants in the LL. After every experiment and after the LL as a whole the results were evaluated. A list of the general types of incentives, as identified by the survey and through their use in the LLs is given in Table 5.

**Table 5:** List of proposed incentives and their implementation in the Living Labs.

Incentive	LL Enschede	LL Gothenburg	LL Leeds
(1) Real-time travel information provided by the system	Was not incorporated in the system		
(2) Social networks for peer-to-peer travel info.	Included in LL setting	Included in LL setting	Included in LL setting
(3) Feedback based on self-monitoring of own travel behaviour	Included in LL setting	Included in LL setting	Included in LL setting
(4) Feedback based on setting targets	Was not incorporated in the system		
(5) Challenges (using points without an exchange value)	Included in experiment setting: Changing departure time; Stimulating cycling	Included in experiment setting: Changing departure time; Changing travel modes	
(6) Challenges (using points with an exchange value)	Included in experiment setting: Stimulating cycling		
(7) Social networks for sharing location	Included in LL setting		Included in Focus group evaluation
(8) Social networks for finding a buddy	Included in LL setting	Included in LL setting	Included in Focus group evaluation
(9) Social networks for treasure hunt	Included in Focus group evaluation		

**Figure 9:**
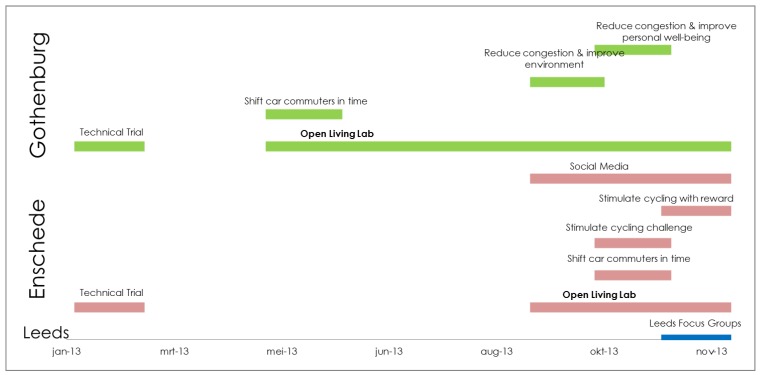
Timeline of experiments in Enschede, Gothenburg and Leeds.

The timeline of the different internal and external experiments and evaluations is shown in Figure 9. Tripzoom was available in the app stores from the start of 2013. From that time forward, people could install and use tripzoom and register their trips. However, only when explicitly recruiting people participants started using tripzoom and users are only prone to use tripzoom while an experiment is conducted (see Section 5 for further comments about this).

During the LL operation, the experimental design used focused experiments with a specific research focus and targeted at a specific group of LL participants. All participants were placed in one group and a selection from this group was made when an experiment was executed. This was conducted using the relevant CD (City Dashboard) functionality. In this way, we are able to estimate the general contribution of tripzoom to a change in behaviour and attitude and the effects of specific incentives to the selected target group. This approach offered us flexibility in the design and operation of the experiments and enabled us to exploit our limited resources to the fullest. 

The experiments more specifically also focussed on evaluating the effectiveness of a challenge and reward type incentives, see Table 6. The first experiment evaluated the DepTimeCar challenge to shift the departure time and was focused on incentivising car drivers in Enschede and Gothenburg to change their departure time to avoid travelling during peak hours. In both these LLs, participants were selected to receive the incentive based on the registered travel behaviour in the familiarisation phase. 

The second experiment called: “reduce congestion and improve environment” was offered in Gothenburg LL. In this experiment all users received a challenge. In total, two challenges were introduced to users, one challenge called DepTimePT focussed on avoiding peak hours for public transport users and one challenge called BusBike focussed on a transport mode shift from car to more sustainable modes of transportation such as travel by bike or bus. 

The third experiment “reduce congestion to improve personal well-being” was offered in Gothenburg LL. Two challenges were conducted with newly recruited users through the Gothenburg Metro newspaper. The ModeDepTime challenge focussed on the environmental protection and congestion objective of SUNSET and rewarded users that used either bus or tram outside the defined peak hour (7:00 to 8:00). This challenge was targeted at car users. The bicycle challenge focussed on both the environmental protection and personal well-being objective. It rewarded current public transport users if they travelled by bicycle instead. The increased use of bicycle is promoted for improving personal well-being. The final BicyclePie challenge was aimed at all travellers to assess if offering a tangible reward, a pie, increased the use of bikes thus improving personal well-being.

**Table 6:** Summary of different types of incentive challenge issued during the LL evaluations and the proposed points reward for meeting the challenge (The two numbers of users are the number who participated in the challenge, with the number who met the challenge given in brackets).

Incentive	Living Lab	SUNSET Objective	Reward	Users
DepTimeCar	Enschede	Congestion	100 points	15(3)
DepTimeCar	Gothenburg	Congestion	500 points	25(7)
DepTimePT	Gothenburg	Congestion	1000–1500 points	10(4)
BusBike	Gothenburg	Environment	500–750 points	15(7)
ModeDepTime	Gothenburg	Congestion + Environment	500–1000 points	10(2)
Bicycle	Gothenburg	Environment + Well-being	500–1000 points	10(6)
Bicycle	Enschede	Environment + Well-being	100 points	22(11)
BicyclePie	Enschede	Environment + Well-being	Pie	50

The evaluation of the behavioural change in the experiments was primarily based on qualitative questionnaires prior to, during, and after the LLs with the quantitative travel behaviour data collected by the tripzoom app, used to support the findings from the questionnaires. The quantity and quality of the travel behaviour data collected by tripzoom meant that a “light” application of the evaluation framework was most appropriate, concentrating on a small number of key indicators, such as the time of departure, travel time and perceptions of the system. This mixed-method approach provided both the observed behaviour, *i.e.*, from tripzoom, and the user characteristics and rationale, *i.e.*, from questionnaires. This approach enabled this study to fully exploit the input and data of the limited number of LL participants and to measure within-subject changes that suggest macro-level effects in the different cities in case of a larger up-take of the tripzoom app.

### 4.3. Results

*Flexibility or ability to reduce congestion*: In total, 77% of the participants in the Enschede LL report that they have flexible working hours. However, when asked if are you willing to shift your departure time outside peak hours, only 23% of the respondents indicated they were willing to commute after peak hours, *i.e.*, only a minority have a predisposition to reducing congestion.

*Accuracy of trip detection and classification*: it is essential that in order for incentive challenges to be accurately targeted that trips need to be accurately detected. A prior technical trial involving 60 users, which undertook 601 trips, was undertaken before the specific incentive-driven experiment trials began, to assess registering the timing of the trip in combination with the trip registration and transport modality detection. It was found that only 28% of all trips were considered as full registrations or completely. About 50% of trips were registered but were not fully correct in modality, departure time or travel time. In the comments 8% of the trips showed comments that the trip was either too short or too long. In 4% of the cases a trip was registered, however no trip was made, a so-called “ghost trip”. 

*Incentive (DepTimeCar) to challenge car users to shift departure time to reduce congestion*: In Enschede over 50% of the target group in the experiment reported that the challenge was not relevant and not feasible to them. Less than 30% of the participants indicated the challenge was relevant and less than 20% indicated the challenge was feasible. This is in line with the reported travel predisposition in LL to travel. 18% of the selected participants for the challenge actually accepted the challenge. Across both Enschede and Gothenburg about 15% of people actually changed the departure times.

*Incentive (DepTimePT) to challenge users to avoid peak hour use of public transport*: due to technical problems this challenge produced no useful results.

*Incentive (BusBike) to challenge users to reduce congestion and improve environment via a transport mode shift to bus or bicycle*: Little over half of the respondents (56%) answered that the challenge was useful, while 38% did not think the challenge was useful. A meaningful challenge depends to a large extent on personal circumstances.

*Incentive (Bicycle) to challenge users to stimulate cycling was:* this was conducted in Enschede*:* 50% of participants agreed the challenge was relevant to them and 67% of participants considered the challenge to be feasible. The challenge to cycle more is more appealing to users in Enschede than the challenge for car drivers to change departure time (DepTimeCar) because the share of the bike in commuting is higher. Moreover also cycling for other purposes could be considered as relevant and feasible to users. 60% of the target group accepted the challenge. However, none of the participants indicated that they had increased the number of bicycle trips during peak hours. This could be caused by the situation that if participants cycle to work or school they cannot make any more trips by bike.

*Incentive (BicyclePie*) *to offer a tangible reward for wellbeing travel (bicycle trips)*: all registered users in the Enschede LL received a challenge over a two week period to cycle 6 kilometres and receive a tasteful reward. After completing the challenge, the user got a pie as a reward. Via e-mail they could indicate when and where they would like to pick the pie as a reward up. The LLc then informed the pie shop about the reservations and informed the user about the location of the actual shop to pick it up. 34% of users met the challenge and retrieved the pie. 46% of the users made the bicycle trip. 28% of users were reported to have received a notification for having completed the challenge. However, of these users that completed the challenge, only 36% actually made the effort to retrieve the tangible reward to collect the pie to eat!

## 5. Conclusions and Outlook

### 5.1. Conclusions about the Experience of Using Incentives to Shift Travel Behaviour

The main conclusion concerning the outcome of experiments to evaluate the use of a challenge and rewards type incentive is that travellers have the potential to change behaviour but this needs to be individualised and context-based. The level and use of non-tangible rewards does not seem to influence users much to shift their behaviour to act greener. The level of personalisation is an important characteristic for the effectiveness of an incentive. It is feasible to make car drivers change their departure time. It is hard to induce car drivers to change to public transport. It is feasible to induce public transport users to change to cycling. Social network concepts are rated best when these provide information useful for the individual; sharing information and experiences does not seem to contribute to shifting travel behaviour.

### 5.2. Conclusions about the Experience of Technology Support for the Use of Non-Tangible Incentives to Shift Travel Behaviour

The main conclusion is that in order for technology to incentivise shifts in societal behaviour, towards more sustainable travel, it needs to be: open, context-aware (situated, timely, energy-aware), accurate, integrate disparate IoT systems, be usable, and be intelligent to target appropriate uses and users. No single location-determination technology is accurate enough to determine pervasively locations and to classify transport track usage and modes. In early 2011, when the EU SUNSET project started, inertial mobile phone sensing was still in its infancy. The accelerometer mobile phone sensor at that time needed to be a foreground task, using a lit display; this was very power-hungry. As a result, the technologies at that time to classify transport track usage and transport modes relied heavily on techniques that determine positions using GPS, the derived speed from this, and related detected locations and tracts to recognised tracks such as specific public transport routes. However, the accuracy of a unilateral GPS approach, even if combined with GIS support, is not high and it is often not that energy efficient. More recently, work has highlighted that inertial mobile phone sensing, based upon accelerometer, can operate in a more energy-efficient way, can be combined with GPS and lead to more accurate, and even more energy-efficient location determination and transport mode recognition [37]. Many systems rely on the use of a remote, e.g., cloud-based, data processing service to classify data, but this incurs a significant latency cost, energy-cost, and hinders time-critical transport mode and route recognition; leveraging, local mobile device inertial location sensing and local data processing can overcome this [38]. Incentives-based systems are essentially a type of event-driven—more specifically, an event-condition-action—rule-based, system. While it is possible to pre-determine some rules, the key stakeholders such as the transport authority rule setters often require rules to be flexible, context-aware and to be able to prescribe new types of rules once the system is in operation. This requires the design and implementation of a more flexible rule-based system [39]. It is exceedingly difficult to build and offer targeted incentives using a single source of information. At the project onset, early 2011, no a priori ODI (Open Data Initiative) data agreements existed in order to access real-time traffic monitoring data feeds in any of the three LL cities as part of the project and to be able to fuse them with the mobile device data. The data agreements for access to the fixed sensor, e.g., traffic light data were useful, i.e., to develop predictive road congestion state prediction service, but such fixed sensor data could only be accessed in a non-real-time, historical mode [40]. Providing the technological support system for incentive-driven mobility, accurate, context-ware information is important in order to facilitate actionable sustainable transport mode shifts. 

### 5.3. Outlook

Using a range of experiments during the LL operation, SUNSET has showcased that individual behavioural change can be achieved in terms of changes in travel times (“time-shifted” out of rush hour) and changes in modality (shifted towards sustainable bike alternatives). In the analysis, a unique combination of both quantitative personal-level travel data analysis and qualitative user evaluation was performed. However, despite promotional activities, tripzoom was not successful as a commercial level attractive, seamless service to end-users that led to broad take-up. As a result, the SUNSET project as a consortium was not able to have the envisioned city level effects on congestion reduction, increased safety, sustainable travel and personal well-being. 

However, tripzoom did deliver a basic personal mobility measurement engine on which public or private stakeholders like local or regional public authorities, employers, transport companies, local retailers, service providers and event organisers can couple, integrate or build their own (cross-sectoral) services, Apps and campaigns, on a scale of their choice to incentivise travellers (commuters, local citizens, visitors, tourists). There exists potentially a fast growing market, as tripzoom types of services are flexible and cost-effective opposed to more traditional traffic- or mobility-management solutions, and allow for new business models. For example, they can facilitate information exchange and incentive distribution between third parties, city authorities, transport suppliers, employers and end-users.
